# COVID-19-related disruptions and measles vaccination uptake among children attending urban health centers in Guelmim, Morocco: a cross-sectional study

**DOI:** 10.1186/s12889-026-28209-z

**Published:** 2026-06-25

**Authors:** Abdelghani Asraoui, Abdelmajid Soulaymani

**Affiliations:** 1 Laboratory of Biology and Health, Faculty of Science, Ibn Tofail University, Kenitra, B.P. 242 Morocco; 2 Anti-Poison and Pharmacovigilance Center, Rabat, Morocco

**Keywords:** Measles, COVID-19, Routine immunization, Vaccine confidence, Catch-up vaccination, Morocco

## Abstract

**Background:**

The COVID-19 pandemic disrupted routine immunization services and may have widened measles immunity gaps. Morocco has experienced a widespread measles outbreak since late 2023. This study assessed the association between COVID-19-related disruptions and measles vaccination uptake among children in Guelmim, Morocco, and described additional informational and access barriers.

**Methods:**

A descriptive cross-sectional study was conducted from March to May 2025 in three urban public health centers in Guelmim. Using an interviewer-administered questionnaire, data were collected from 350 mothers of children aged 0–59 months.

Descriptive statistics and Pearson’s chi-square tests were used to examine associations between child measles vaccination status and COVID-19-related, informational, and access-related factors.

**Results:**

Only 44.29% of children were reported as vaccinated against measles. Lower uptake was significantly associated with delaying child vaccination during COVID-19 (27.27% vaccinated vs. 69.61% among those reporting no delay; *p* < 0.001), missing a measles dose during the pandemic (28.64% vs. 67.96%; *p* < 0.001), insufficient vaccine information (28.19% vs. 73.98%; *p* < 0.001), lack of trust in vaccines offered by the public health system (28.29% vs. 68.92%; *p* < 0.001), difficulty accessing vaccination services (35.88% vs. 69.32%; *p* < 0.001), and lack of information on post-pandemic vaccination dates (25.00% vs. 68.83%; *p* < 0.001). Receipt of a measles catch-up dose was strongly associated with vaccination uptake (82.79% among those receiving catch-up; *p* < 0.001).

**Conclusions:**

In this urban sample, measles vaccination uptake was low and was associated with both COVID-19-related service disruptions and broader informational, trust, and access barriers. Strengthening catch-up activities, service communication, and trust-building strategies may help close immunity gaps in similar settings.

**Supplementary Information:**

The online version contains supplementary material available at https://doi.org/10.1186/s12889-026-28209-z.

## Introduction

Measles remains one of the most contagious vaccine-preventable diseases and continues to threaten child health despite the availability of a safe and highly effective vaccine [1, 2].

Sustained population protection requires high two-dose coverage, because even modest declines in vaccination uptake can rapidly create immunity gaps and enable outbreaks [1, 2].

The COVID-19 pandemic interrupted immunization services worldwide through movement restrictions, service reorganization, supply interruptions, and fear of exposure in health facilities [3–5]. These disruptions slowed or reversed progress in routine childhood vaccination in many countries, especially where health systems already faced communication, access, or trust challenges [4, 5].

In Morocco, measles vaccination is provided through the national immunization program in a two-dose schedule, with the first dose at 9 months and the second between 15 and 18 months [6]. Since late 2023, Morocco has experienced a widespread measles outbreak affecting all 12 regions; by 13 April 2025, more than 25,000 suspected cases, 13,706 laboratory-confirmed cases, and 184 deaths had been reported [6]. At the same time, official sources emphasize that the 95% coverage threshold needed to sustain elimination has not been achieved uniformly in recent years [6, 7].

The Moroccan experience during the pandemic also suggests pressure on routine childhood immunization services. An earlier survey of Moroccan pediatricians described a substantial decline in childhood vaccination during the COVID-19 period and called for corrective measures to restore routine vaccination activities [8]. However, local evidence from caregivers remains limited, particularly in southern urban settings such as Guelmim, where service access and public confidence may vary substantially.

This study therefore aimed to assess the association between COVID-19-related disruptions and measles vaccination uptake among children under five attending urban health centers in Guelmim, while also documenting broader informational, attitudinal, and access-related factors that may shape vaccination behavior in the post-pandemic period.

## Methods

### Study design and setting

A descriptive cross-sectional survey was conducted between March and May 2025 in the city of Guelmim, southern Morocco. Data were collected in three urban public health centers-Amhayrich, Tayert, and Gramez-which provide routine maternal and child health services, including childhood vaccination. These facilities were selected because they are routine urban entry points for immunization services in Guelmim. The study was not designed to yield a statistically representative regional estimate; rather, it provides evidence from a facility-based urban sample.

### Study population and sampling

Eligible participants were mothers aged 18 years or older who were the primary caregivers of at least one child aged 0–59 months, had resided in Guelmim for at least 12 months, and attended one of the selected facilities during the study period. Mothers with major cognitive impairment affecting comprehension or recall, and temporary visitors to the area, were excluded. A total of 350 mothers were enrolled using convenience sampling, an approach commonly used in facility-based surveys when the target population is encountered in routine care settings [9].

### Questionnaire and study variables

Data were collected using a structured interviewer-administered questionnaire adapted from WHO vaccine coverage guidance and WHO/UNICEF vaccine hesitancy frameworks [10, 11].

A pilot test with 15 mothers was used to improve wording and contextual relevance. The questionnaire covered five domains: (1) sociodemographic characteristics; (2) maternal COVID-19 vaccination history; (3) the child’s measles vaccination status and timing; (4) information sources, trust in vaccines, vaccine concerns, and history of vaccine refusal; and (5) access to services, campaign participation, and awareness of catch-up activities. To better reflect the study objective, variables were grouped in the revised manuscript into COVID-19-related factors and broader non-COVID determinants of uptake.

### Ethical considerations

Administrative authorization to access the selected health centers was obtained from the Regional Directorate of Health in Guelmim. The study was conducted in accordance with the ethical principles of the Declaration of Helsinki. Ethical approval was granted by the Biomedical Research Ethics Committee of Rabat (approval No. 89/21). Written informed consent was obtained from all participants prior to the interviews. No personally identifying information was recorded, and all responses were analyzed anonymously.

### Statistical analysis

Data entry and cleaning were conducted in Microsoft Excel 2016 and statistical analyses were performed in IBM SPSS Statistics version 27.0. Descriptive statistics are presented as frequencies and percentages. Associations between the child’s measles vaccination status (vaccinated vs. non-vaccinated) and explanatory variables were assessed using Pearson’s chi-square test. Statistical significance was set at *p* < 0.05. Because the study is cross-sectional and based on self-reported information, the findings are interpreted as associations rather than causal effects.

## Results

Among the 350 surveyed mothers, the largest age group was 30–39 years (54.86%), followed by 20–29 years (30.29%). Nearly half had primary education (48.85%), 54.29% were housewives, and 50.86% reported having two children (Table 1).

**Table 1:** Sociodemographic characteristics of mothers (*N* = 350)

Variable	Category	*n*	%
Mother’s age (years)	Under 20	21	6.00
	20–29	106	30.29
	30–39	192	54.86
	40–49	27	7.71
	50 or older	4	1.14
Education level	No schooling	36	10.29
	Primary	171	48.85
	College	70	20.00
	Secondary	37	10.57
	Higher education	36	10.29
Occupation	Housewife	190	54.29
	Civil servant	34	9.71
	Private sector	38	10.86
	Self-employed	58	16.57
	Unemployed	24	6.86
	Other	6	1.71
Number of children	1	62	17.71
	2	178	50.86
	3	80	22.86
	4 or more	30	8.57

Percentages are calculated using *N* = 350 and displayed to two decimal places

Vaccination-related behaviors and perceptions suggested an environment marked by information and trust constraints. Although 69.14% of mothers had received at least one dose of a COVID-19 vaccine, 59.71% reported postponing or missing a child vaccination during the pandemic, only 35.14% considered themselves sufficiently informed about vaccines, and social media was the most frequently cited source of information (42.86%). Trust in vaccines offered by the public health system was low, with 58.57% reporting no trust. Only 44.29% of children were reported as vaccinated against measles, while 60.86% had missed at least one measles dose during the pandemic (Table 2).

**Table 2:** Vaccination-related behaviors, information sources, and measles/COVID-19 perceptions (*N* = 350)

Variable	Category	*n*	%
Maternal COVID-19 vaccination	Vaccinated	242	69.14
	Not vaccinated	108	30.86
Child vaccination delayed during COVID-19	Yes	209	59.71
	No	102	29.14
	Cannot remember	39	11.15
Self-reported change in attention to vaccination after COVID-19	More attentive	241	68.86
	Less attentive	71	20.29
	No change	38	10.85
Sufficient vaccine information	Yes	123	35.14
	No	227	64.86
Main source of vaccine information	Social media	150	42.86
	Health professionals	72	20.57
	Family or friends	70	20.00
	Television/radio	44	12.57
	Internet (other than social media)	14	4.00
Trust in vaccines offered by public health system	Yes	74	21.14
	Partial	71	20.29
	No	205	58.57
History of vaccine refusal	Yes	259	74.00
	No	91	26.00
Main vaccine concern	Lack of information	173	49.43
	Distrust in pharmaceutical companies	60	17.14
	Perceived ineffectiveness	51	14.57
	Fear of side effects	47	13.43
	No concern	19	5.43
Child measles vaccination status	Vaccinated	155	44.29
	Non-vaccinated	195	55.71
Age at measles vaccination	Under 1 year	67	19.14
	1–2 years	80	22.86
	Older than 2 years	8	2.29
	Not applicable (non-vaccinated)	195	55.71
Missed measles vaccine during pandemic	Yes	213	60.86
	No	103	29.43
	Do not remember	34	9.71
Perceived measles as serious	Yes	253	72.29
	No	85	24.28
	Do not know	12	3.43
Observed measles resurgence locally	Yes	268	76.57
	No	66	18.86
	Do not know	16	4.57
Belief that COVID-19 delays contributed to resurgence	Yes	253	72.29
	No	62	17.71
	Do not know	35	10.00
Participation in vaccination campaigns	Yes	245	70.00
	No	105	30.00
Informed of post-pandemic measles vaccination dates	Yes	154	44.00
	No	196	56.00
Received measles catch-up dose	Yes	122	34.86
	No	228	65.14
Difficulty accessing vaccination center	Yes	262	74.86
	No	88	25.14
Need more information about vaccination	Yes	295	84.29
	No	55	15.71

Percentages are calculated using *N* = 350 and displayed to two decimal places

To avoid conflating pandemic-specific and broader determinants, the bivariate results are presented in two separate tables. Table 3 summarizes COVID-19-related factors, whereas Table 4 presents informational, attitudinal, and access-related factors. 

**Table 3:** COVID-19-related factors associated with child measles vaccination status (*N* = 350)

Factor	Category	Non-vaccinated *n* (%)	Vaccinated *n* (%)	*p*-value
Mother vaccinated against COVID-19	No	52 (48.15)	56 (51.85)	0.057
	Yes	143 (59.09)	99 (40.91)	
Child vaccination delayed during COVID-19	Do not remember	12 (30.77)	27 (69.23)	< 0.001
	No	31 (30.39)	71 (69.61)	
	Yes	152 (72.73)	57 (27.27)	
Change in attention to vaccination after COVID-19	No change	14 (36.84)	24 (63.16)	< 0.001
	Less attentive	25 (35.21)	46 (64.79)	
	More attentive	156 (64.73)	85 (35.27)	
Missed measles dose during pandemic	Do not know	10 (29.41)	24 (70.59)	< 0.001
	No	33 (32.04)	70 (67.96)	
	Yes	152 (71.36)	61 (28.64)	
Belief that COVID-19 delays caused measles return	Do not know	10 (28.57)	25 (71.43)	< 0.001
	No	28 (45.16)	34 (54.84)	
	Yes	157 (62.06)	96 (37.94)	
Participation in vaccination campaigns	No	43 (40.95)	62 (59.05)	< 0.001
	Yes	152 (62.04)	93 (37.96)	
Informed of post-pandemic vaccination dates	No	147 (75.00)	49 (25.00)	< 0.001
	Yes	48 (31.17)	106 (68.83)	
Received measles catch-up dose	No	174 (76.32)	54 (23.68)	< 0.001
	Yes	21 (17.21)	101 (82.79)	

Percentages are calculated within each category of the explanatory variable and displayed to two decimal places

*p*-values were obtained using Pearson's chi-square test

**Table 4:** Informational, attitudinal, and access-related factors associated with child measles vaccination status (*N* = 350)

Factor	Category	Non-vaccinated *n* (%)	Vaccinated *n* (%)	*p*-value
Sufficient vaccine information	No	163 (71.81)	64 (28.19)	< 0.001
	Yes	32 (26.02)	91 (73.98)	
Trust in vaccines offered by public health system	No	147 (71.71)	58 (28.29)	< 0.001
	Yes	23 (31.08)	51 (68.92)	
	Partial	25 (35.21)	46 (64.79)	
Ever refused a vaccine	No	37 (40.66)	54 (59.34)	0.001
	Yes	158 (61.00)	101 (39.00)	
Main concern about vaccines	None	3 (15.79)	16 (84.21)	< 0.001
	Side effects	16 (34.04)	31 (65.96)	
	Effectiveness	19 (37.25)	32 (62.75)	
	Lack of information	136 (78.61)	37 (21.39)	
	Distrust in pharmaceutical companies	21 (35.00)	39 (65.00)	
Perceived measles as serious	Do not know	3 (25.00)	9 (75.00)	0.052
	No	44 (51.76)	41 (48.24)	
	Yes	148 (58.50)	105 (41.50)	
Observed local measles resurgence	Do not know	4 (25.00)	12 (75.00)	0.017
	No	33 (50.00)	33 (50.00)	
	Yes	158 (58.96)	110 (41.04)	
Difficulty accessing vaccination center	No	27 (30.68)	61 (69.32)	< 0.001
	Yes	168 (64.12)	94 (35.88)	
Want more vaccine information	No	23 (41.82)	32 (58.18)	0.024
	Yes	172 (58.31)	123 (41.69)	

Percentages are calculated within each category of the explanatory variable and displayed to two decimal places

*p*-values were obtained using Pearson's chi-square test

### COVID-19-related determinants of measles vaccination uptake

COVID-19-related variables were strongly associated with measles vaccination uptake (Table 3). Children of mothers who reported delaying child vaccination during COVID-19 were much less likely to be vaccinated against measles than those of mothers reporting no delay (27.27% vs. 69.61%; *p* < 0.001). A similar pattern was observed for missing a measles dose during the pandemic (28.64% vs. 67.96%; *p* < 0.001). Coverage was also substantially higher among mothers informed of post-pandemic vaccination dates (68.83% vs. 25.00%; *p* < 0.001) and among those whose child had received a catch-up dose (82.79% vs. 23.68%; *p* < 0.001).

Participation in vaccination campaigns and the belief that COVID-19-related delays contributed to measles resurgence were also significantly associated with vaccination status. Maternal receipt of a COVID-19 vaccine itself was not significantly associated with child measles vaccination (*p* = 0.057). 

### Informational, attitudinal, and access-related determinants

Broader informational, attitudinal, and access-related factors were likewise associated with measles vaccination status (Table 4). Coverage was much higher among mothers reporting sufficient vaccine information (73.98% vs. 28.19%; *p* < 0.001) and among those who trusted vaccines offered by the public health system (68.92% vs. 28.29%; *p* < 0.001). A previous history of vaccine refusal was negatively associated with measles vaccination uptake (59.34% vaccinated among those reporting no prior refusal vs. 39.00% among those reporting refusal; *p* = 0.001). Lack of information was the most frequently cited vaccine concern and corresponded to the lowest vaccination uptake (21.39%; *p* < 0.001). Difficulty accessing vaccination centers was also strongly associated with non-vaccination (35.88% vaccinated among those reporting difficulty vs. 69.32% among those reporting no difficulty; *p* < 0.001).

Perceiving measles as a serious disease showed a borderline association with uptake (*p* = 0.052). 

## Discussion

This revised analysis shows that measles vaccination uptake in this urban sample from Guelmim was low (44.29%) and that lower uptake was associated with both pandemic-related service disruptions and broader structural and informational barriers. The finding is concerning in the Moroccan context because measles transmission intensified nationally after late 2023 despite a long-standing two-dose measles schedule within the national immunization program [6, 7].

The COVID-19 period appears to have been an important context for missed opportunities for vaccination. Mothers who reported delaying child vaccination during COVID-19 or missing a measles dose during the pandemic were substantially less likely to report that their child had been vaccinated. This pattern is consistent with evidence from Morocco and other settings indicating that routine immunization activities were disrupted during the pandemic and required targeted recovery measures [4, 5, 8]. Because the present study is cross-sectional and based on self-report, these findings should be interpreted as perceived and observed associations rather than direct proof of a causal mechanism.

At the same time, the data suggest that the pandemic interacted with barriers that are not unique to COVID-19. The strongest non-pandemic correlates of measles vaccination in this sample were sufficient information, trust in vaccines offered by the public health system, and access to vaccination services. These findings are consistent with broader literature showing that vaccine confidence, service accessibility, and the quality of communication are central determinants of uptake [12–15]. The predominance of social media as a reported information source in this sample does not by itself demonstrate misinformation exposure, but it does indicate that immunization communication competes with informal and potentially unreliable channels and therefore requires proactive public health engagement [14, 15].

The revised presentation also helps distinguish COVID-19-related determinants from more general vaccination determinants. This distinction is important because not all low uptake observed in 2025 can be attributed solely to the pandemic. Broader issues-such as low confidence in public vaccines, prior refusal, and access difficulties-likely predated the pandemic but may have constrained recovery afterwards. In that respect, the Guelmim findings align with literature showing that measles resurgence often reflects a combination of recent service interruption and pre-existing vulnerability in coverage, confidence, or access [6, 13, 16].

Catch-up activities emerged as one of the clearest programmatic signals in this study.

Children who received a measles catch-up dose and mothers who were informed of post-pandemic vaccination dates had substantially higher coverage than their counterparts. This supports the importance of catch-up and supplementary immunization activities in closing immunity gaps after service disruption [17]. In practice, these results suggest that recovery strategies should not rely only on the availability of doses, but also on timely communication, community outreach, and visible efforts to rebuild confidence in routine services.

Several limitations should be considered. First, the cross-sectional design precludes causal inference. Second, all data were self-reported and vaccination status was not verified against immunization cards or health records. Third, the convenience sample was drawn from urban public health centers and cannot be generalized to all mothers in Guelmim or to rural populations. Finally, the questionnaire included both pandemic-specific variables and broader vaccination perception variables; this was useful for understanding the post-pandemic context, but it also means that the study addresses measles vaccination uptake more broadly than COVID-19 effects alone.

## Conclusion

In this facility-based urban sample from Guelmim, measles vaccination uptake among children under five was suboptimal and was associated with both COVID-19-related disruption indicators and broader barriers related to information, trust, and service access.

The findings support strengthening catch-up vaccination, improving communication on vaccination schedules, and investing in confidence-building strategies rooted in routine primary care and community outreach. Future work using verified immunization records and broader sampling frames would help clarify the relative contribution of pandemic-related and long-standing determinants. 

## Supplementary Information

Supplementary Material 1.

## Data Availability

Data supporting the findings of this study are available, upon request from the main author A.A.
