# Supplementary material for: COVID-19-related disruptions and measles vaccination uptake among children attending urban health centers in Guelmim, Morocco: a cross-sectional study
Source: BMC Public Health. 2026 Jun 25;26:2802. doi: 10.1186/s12889-026-28209-z (PMC13637358; doi:10.1186/s12889-026-28209-z)
Supplement: Supplementary file 1 [file 12889_2026_28209_MOESM1_ESM.docx]

**Cover letter - BMC Public Health**

Dear Editor,

Please find attached the revised version of our manuscript entitled "COVID-19-related disruptions and measles vaccination uptake among children attending urban health centers in Guelmim, Morocco: a cross-sectional study" for consideration in BMC Public Health.

We sincerely thank you and the reviewers for the detailed and constructive comments. In response to the major revision decision, we have substantially revised the manuscript. The main changes include:
- revision of the title to better reflect the cross-sectional design and avoid causal overstatement;
- substantial shortening and rewriting of the Introduction;
- clearer description of the Moroccan context, including the national measles vaccination schedule and the recent outbreak;
- clarification of the study setting, sampling approach, and ethics statement;
- reorganization of the Results into COVID-19-related and non-COVID-related determinants;
- splitting and simplification of the tables for improved readability;
- complete rewriting and shortening of the Discussion;
- standardization of the references; and
- addition of a transparent statement on AI-assisted manuscript revision.

No new experiments were conducted because the study is a cross-sectional survey based on an existing dataset. However, the presentation, structure, and interpretation of the findings were substantially improved.

All authors have reviewed and approved the revised manuscript and agree with its resubmission. The manuscript is original, has not been published previously, and is not under consideration elsewhere.

Thank you for your consideration.

Sincerely,

Abdelghani Asraoui
On behalf of all authors
